# Molecular Dynamics-Assisted Discovery of Novel Phosphodiesterase-5 Inhibitors Targeting a Unique Allosteric Pocket

**DOI:** 10.3390/molecules30030588

**Published:** 2025-01-27

**Authors:** Weihao Luo, Runduo Liu, Xinlin Cai, Qian Zhou, Chen Zhang

**Affiliations:** 1 School of Chemistry and Chemical Engineering, Guangdong Pharmaceutical University, Zhongshan 528458, China; 2112373045@stu.gdpu.edu.cn (W.L.); 2112440011@stu.gdpu.edu.cn (X.C.); 2 School of Pharmaceutical Sciences, Sun Yat-sen University, Guangzhou 510006, China; liurd3@mail2.sysu.edu.cn; 3 Key Laboratory of Tropical Biological Resources of Ministry of Education, Hainan Engineering Research Center for Drug Screening and Evaluation, School of Pharmaceutical Sciences, Hainan University, Haikou 570228, China

**Keywords:** phosphodiesterase-5, allosteric pocket, selectivity, molecular dynamics

## Abstract

Phosphodiesterase-5 (PDE5) is a potent therapeutic target for the treatment of male erectile dysfunction and pulmonary arterial hypertension with several drugs available on the market. However, most of the reported PDE5 inhibitors lack specificity over PDE6, a holoenzyme in eleven PDE families, which may cause various adverse effects. Targeting a unique allosteric pocket has proved to be an effective approach to designing selective PDE5 inhibitors. In the present study, an integrated virtual screening procedure consisting of pharmacophore modeling screening, molecular docking, molecular dynamics simulations, and binding free energy calculations was applied to the discovery of novel PDE5 inhibitors targeting the allosteric pocket. Seven out of thirty-three molecules purchased from the SPECS database (a hitting accuracy of 21%) with novel scaffolds were PDE5 inhibitors with enzymatic inhibition ratios of more than 50% at a concentration of 10 μM. Predicted binding patterns indicate these hits fit well in the allosteric pocket in PDE5. In particular, compound AI-898/12177002 (IC_50_ = 1.6 μM) demonstrates over 10-fold selectivity towards PDE6, providing a novel scaffold for the optimization of potent and selective PDE5 inhibitors with less adverse effects.

## 1. Introduction

Cyclic adenosine monophosphate (cAMP) and cyclic guanosine monophosphate (cGMP) act as the second messengers in cellular signaling pathways. The cAMP and cGMP play important roles in mediating broad biological processes; for instance, cell proliferation and differentiation, smooth muscle relaxation and vasodilatation, immune and inflammation reactions, and memory [1,2]. Phosphodiesterases (PDEs) are the sole enzymes in charge of hydrolyzing the cAMP and cGMP, and thus are potential drug targets for various diseases [3,4,5]. Currently, several successful drugs targeting a single PDE are common in clinical usage, such as PDE4 inhibitor apremilast for the treatment of psoriatic arthritis, and PDE3 inhibitor cilostazol for the treatment of chronic arterial occlusion [5,6,7].

Among the eleven PDE families, PDE5, 6, and 9 exclusively hydrolyze cGMP into its inactive 5′-GMP form. Structurally, PDE5 functions as a homodimer, while each monomer possesses an N-terminal regulatory domain and a C-terminal catalytic domain [8,9]. The N-terminal contains two GAF (cGMP-binding and activation of PDEs) domains capable of regulating the enzymatic activity of the catalytic domain [10]. The catalytic domain in the C-terminal is structurally conserved across PDE families, which consists of several invariant residues such as phenylalanine, glutamine, as well as histidine and carboxylate (aspartate) essential for metal ion binding [11]. PDE5 is a key enzyme in NO-sGC-cGMP-PKG signaling pathways, with wide distribution in smooth muscle, platelets, the lungs, kidneys, and heart [12,13]. This distribution makes PDE5 a key contributor to the regulation of pivotal physiological processes, including smooth muscle relaxation, vasodilation, and platelet function [8,14].

**Figure 1:**
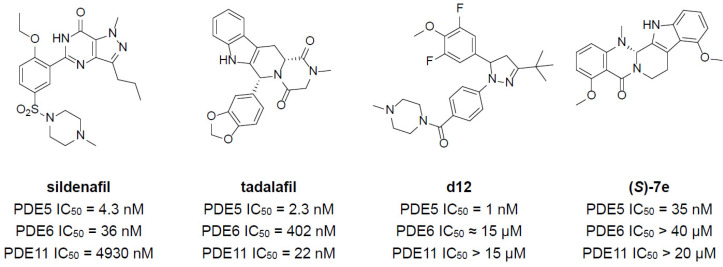
Representative PDE5 inhibitors.

Inhibition of PDE5 has been validated to enhance cGMP levels in specific tissues and benefit patients with various diseases such as male erectile dysfunction and pulmonary arterial hypertension [15,16,17]. The therapeutic potential of PDE5 inhibitors has led to the development of several drugs with the most well-known ones being sildenafil citrate and tadalafil (Figure 1) [18,19]. However, most of the reported PDE5 inhibitors lack selectivity over PDE6 or PDE11 and may cause unneglectable side effects [20,21]. This is due to the extreme similarity of the catalytic domains of PDE5 and PDE6/PDE11 where competitive inhibitors bind (sequence similarities exceeding 50%) [22]. Many PDE5 inhibitors exhibit similar pharmacophore features to PDE6 ones, such as a purine-like fused ring and alkoxybenzene moiety, leading to concurrent inhibition against PDE6. Using these PDE5 inhibitors with limited selectivity can result in off-target inhibition of PDE6 in the retina, which disrupts visual processes, or PDE11 in muscle tissue, leading to muscle pain and other systemic effects. For example, administration of sildenafil frequently causes visual disturbances, facial flushing, or headaches. Administration of tadalafil frequently causes back and muscle pains [23]. Recently, Abdel-Halim and coworkers developed a novel PDE5 inhibitor **d12** based on celecoxib, with over 10,000-fold selectivity over PDE6 and PDE11 [24]. The **d12** was deduced to bind to the interface region between the regulatory/catalytic domain or an allosteric site on the regulatory domain. At the same time, novel evodiamine derivatives were synthesized and validated to be highly selective PDE5 inhibitors by Zhang and coworkers [25]. Among them, **(*S*)-7e** demonstrated over 500-fold selectivity against PDE6 and PDE11. X-ray crystal structure research discovered a unique allosteric pocket in the C-terminal of PDE5 with bound **(*S*)-7e** for the first time. Thus, targeting this allosteric pocket provides a novel means of designing and developing PDE5 inhibitors with minimal side effects caused by simultaneous inhibition of PDE6/PDE11.

**Figure 2:**
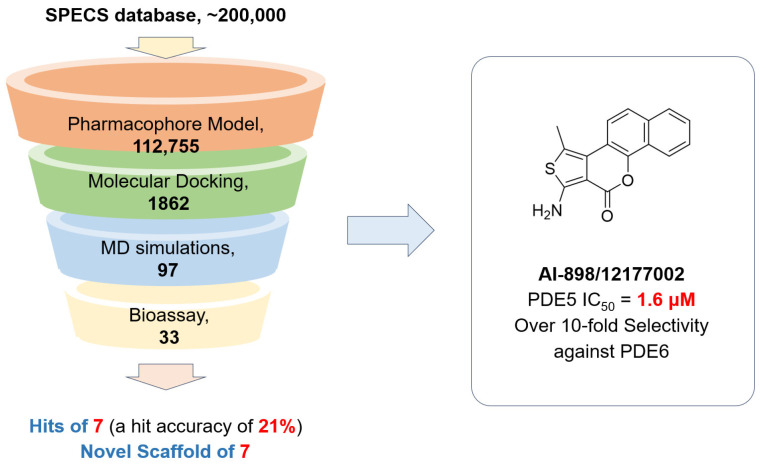
Workflow of the virtual screening processes, combining the pharmacophore model screening, molecular docking, MD simulations, and bioassays.

With our continuous interest in the discovery of novel PDE inhibitors [26,27,28], herein we applied an established virtual screening method combining pharmacophore modelling, molecular docking, and molecular dynamics simulations to discover novel PDE5 inhibitors targeting the unique allosteric pocket in the catalytic domain. Seven out of thirty-three molecules purchased from the SPECS database (a hitting accuracy of 21%) with novel scaffolds were validated as PDE5 inhibitors with more than 50% enzymatic inhibition ratios at a concentration of 10 μM. In particular, compound **AI-898/12177002** (IC_50_ = 1.6 μM) demonstrates over 10-fold selectivity towards PDE6 (Figure 2).

## 2. Results

### 2.1. Pharmacophore Modelling and Screening Based on Cocrystal Structure of PDE5 and **(S)-7e**

**Figure 3:**
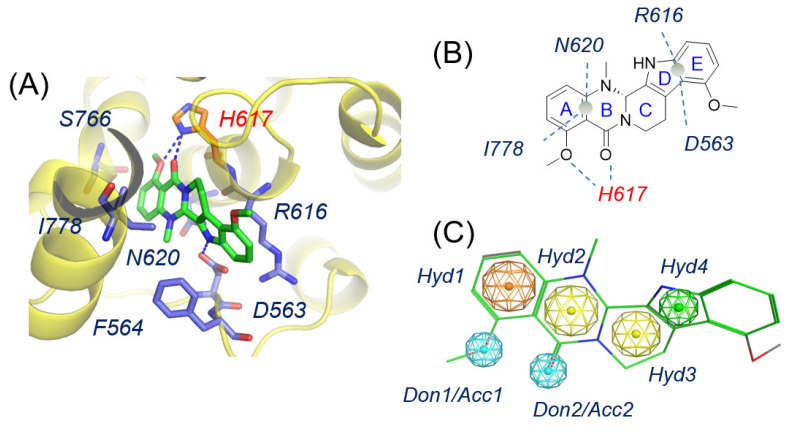
Binding pattern of **(*S*)-7e** with PDE5 (**A**,**B**) and established pharmacophore model (**C**). His617 was deduced as a key residue for the binding of the allosteric inhibitor, which is labeled in red.

The co-crystal structure of the PDE5 catalytic domain complexed with **(*S*)-7e** (PDB ID: 6VBI, Figure 3A) [25] provides valuable insights into the proposed binding patterns of inhibitors in the allosteric pocket: (1) The allosteric pocket features a narrow opening and a relatively deep internal space. (2) Inhibitors entering the pocket should have a rigid or planar structure with minimal side chains. (3) Conjugated ring structures are crucial, with multiple substituents on the rings that form hydrogen bonds with His617 and Asp563.

Based on the binding patterns, a pharmacophore model was initially developed (see Figure S1), incorporating representative features and carefully refined to prevent excessive compound matches (see Supporting Information). The final pharmacophore model included (1) two key features representing conjugated hydrophobic ring centers, labeled Hyd1 and Hyd2; (2) two alternative features for conjugated hydrophobic ring centers, labeled Hyd3 and Hyd4; and (3) two hydrogen bonding-related features that act as hydrogen bond donors or acceptors, labeled Don/Acc (Figure 3B,C). During pharmacophore screening, three out of four features representing hydrophobic centers were selected, and a binary choice was made between the hydrogen bond donor/acceptor features for partial matching.

Comprehensive conformational sampling for each compound in the SPECS database was performed before pharmacophore screening. Filtration based on “Rule of Five” [29] was simultaneously applied during conformation generation to exclude compounds lacking drug-like properties. By standardizing bonding parameters, including bond lengths, angles, and dihedrals, as well as rotating rotatable bonds, up to 255 conformations were generated for each compound, resulting in **Dataset 01** with 112,755 compounds. These conformations were then screened using the established pharmacophore model. A total of 13,620 compounds with diverse conformations passed the pharmacophore screening, forming **Dataset 02**. To further refine the dataset, the conformations in **Dataset 02** were filtered using the pharmacophore model with excluded volume constraints based on neighboring residues to prevent steric hindrance with the allosteric pocket (see Supporting Information). This step significantly reduced the dataset size from 13,620 compounds in **Dataset 02** to 1862 compounds in **Dataset 03**.

### 2.2. Molecular Docking Screening for Appropriate Binding Patterns Between Compounds and the Allosteric Pocket of PDE5

To prevent interference with subsequent screening steps, pan assay interference compounds (PAINS) were removed from the dataset using the online tool ’PAINS-remover’ (https://www.cbligand.org/PAINS, accessed on 9 January 2024) before molecular docking [30]. All compounds in **Dataset 04**, with PAINS removed from **Dataset 03**, were then subjected to molecular docking screening to predict their binding patterns with the allosteric pocket of PDE5 and further reduce the dataset size.

In the allosteric pocket of the C-terminal of PDE5, the A and B rings of **(*S*)-7e** were sandwiched between Leu778 and Asn620, forming hydrogen bonds with His617 via its carboxyl and methoxyl groups. A similar sandwich-like interaction was observed between the E and F rings of **(*S*)-7e** and Phe564 and Arg616, along with a hydrogen bond to Asp563 (Figure 3A,B). We concluded that hydrogen bonding with His617 plays a crucial role in receptor–inhibitor recognition and allosteric regulation. Therefore, these hydrogen bonding interactions with His617 were preserved during the molecular docking screening process. The top-ranked 97 compounds, selected based on higher docking scores and optimal binding patterns, formed **Dataset 05** for further molecular dynamics studies. The binding modes of representative molecules with PDE5 are shown in Figure S2.

### 2.3. Identification of PDE5 Inhibitors by Molecular Dynamics-Assisted Screening and Bioassay

**Figure 4:**
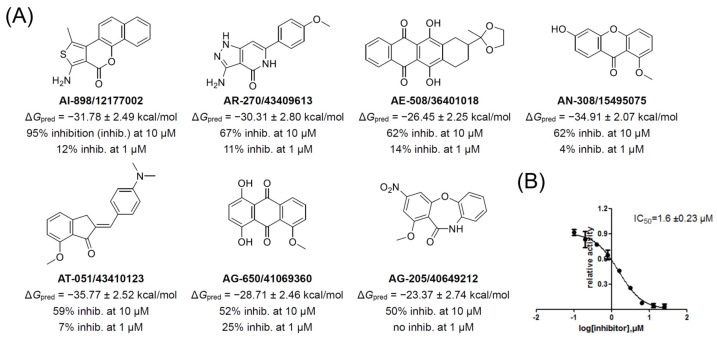
(**A**) Chemical structures of the hits, their predicted binding free energies based on MM-GBSA method (Δ*G*_pred_), and inhibition ratios. (**B**) The inhibitory profile of **AI-898/12177002** with an unambiguous dose-dependent effect.

To screen and obtain structurally stable systems of PDE5 bound to small molecules, molecular dynamics (MD) simulations were performed using Amber 16 [31]. After 20 ns of MD simulations, MM-PBSA and MM-GBSA binding free energy calculations were carried out [32]. Systems with stable trajectory RMSD values (for residue backbone atoms, less than 2.0 Å) and more negative binding free energies were selected, indicating that these compounds exhibited both stable binding patterns and strong binding energies. As a result, 33 compounds were retained to form **Dataset 06**. These 33 compounds were then purchased from SPECS for further bioactivity testing. The predicted binding free energies of these compounds and their inhibition ratios against PDE5 are listed in Table S1. Sildenafil was used as the reference compound, with an inhibition ratio of over 50% at a concentration of 10 nM. Seven of the thirty-three compounds purchased were confirmed to be PDE5 inhibitors, with inhibition ratios exceeding 50% at 10 μM (Figure 4A). Among them, compound **AI-898/12177002** exhibited the highest inhibition ratio at 10 μM, with an IC_50_ of 1.6 μM against PDE5 (Figure 4B). Further enzymatic tests revealed that **AI-898/12177002** showed inhibition ratios of 8%, 79%, and 63% at a concentration of 10 μM against PDE6, PDE10, and PDE11, respectively, indicating that it possesses over 10-fold selectivity towards PDE6 (see Table S1).

## 3. Discussion

### 3.1. New Scaffolds for Hits as PDE5 Inhibitors

**Figure 5:**
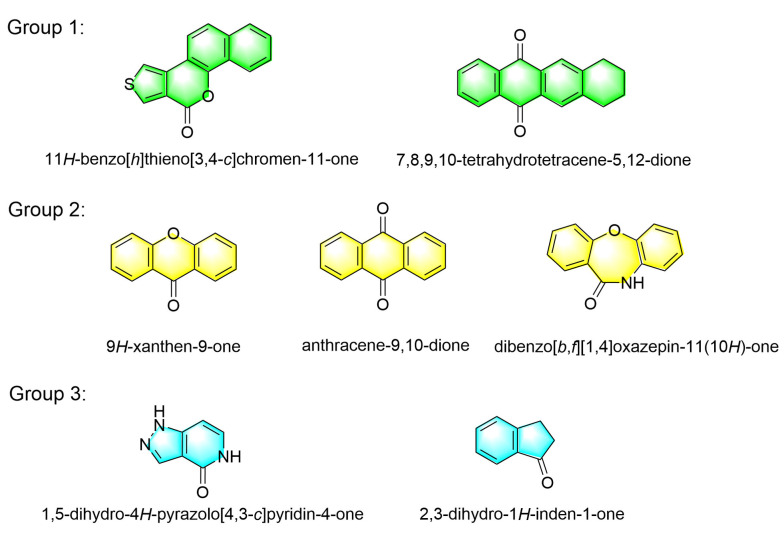
Seven novel scaffolds from hits as PDE5 inhibitors which are divided into three groups.

All hits feature new scaffolds compared to existing PDE5 inhibitors, as indicated by molecular similarity calculations (see Table S2). These seven identified PDE5 inhibitors can be classified into three groups based on the number of fused rings in their scaffolds. Group 1 includes **AI-898/12177002** and **AE-508/36401018**, which contain four fused rings. Group 2 includes **AN-308/15495075**, **AG-650/41069360**, and **AG-205/40649212**, which contain three fused rings. Group 3 consists of **AR-270/43409613** and **AT-051/43410123**, which contain two fused rings (Figure 5).

### 3.2. Binding Patterns and Structure–Activity Relationship of Novel PDE5 Inhibitors

**Figure 6:**
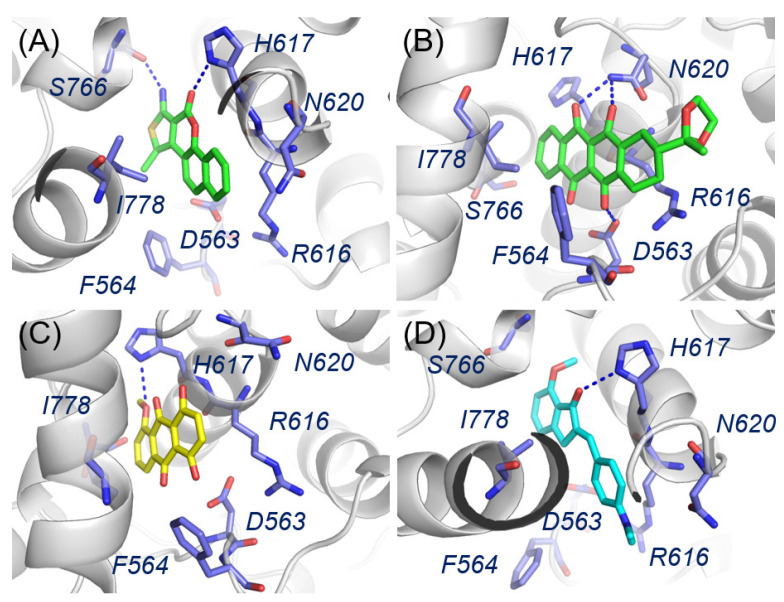
Binding modes of representative inhibitors **AI-898/12177002** (**A**), **AE-508/36401018** (**B**), **AG-650/41069360** (**C**), and **AT-051/43410123** (**D**) within the allosteric pocket in PDE5 C-terminal (PDB ID: 6VBI, color in green, key residues in deep blue) after 20 ns MD simulations. Hydrogen bonds are depicted in blue dashed lines.

The binding modes of the identified PDE5 inhibitors after 20 ns of MD simulations were analyzed to provide insights for structural modifications. The predicted binding patterns of four representative inhibitors are shown in Figure 6. All four inhibitors were positioned within the allosteric pocket, sandwiched between Ile778, Asn620, and Arg616, forming significant hydrophobic interactions that are crucial for binding to PDE5. Additionally, **AI-898/12177002**, **AG-650/41069360**, and **AT-051/43410123** interacted with His617 in the allosteric pocket via hydrogen bonds, similar to the binding modes of **(*S*)-7e** with PDE5. Notably, **AI-898/12177002** was able to form an extra hydrogen bond with Ser766 at the deepest part of the pocket, which may explain its higher inhibition ratio compared to the other hit inhibitors (Figure 6A). **AG-650/41069360** lacks a substituent extending outward from the pocket (Figure 6C), in contrast to **AE-508/36401018** (Figure 6B) or **AT-051/43410123** (Figure 6D). Due to its four-fused-ring scaffold and bulky 2-methyl-1,3-dioxolan-2-yl group, **AE-508/36401018** slightly protruded from the pocket, preventing hydrogen bonding with the key residue His617. Instead, it formed hydrogen bonds with Asn620 and Asp563 (Figure 6D). This suggests that hydrogen bonding interactions with Asn620 or Asp563 could represent a new binding pattern for inhibitors, offering a potential strategy for virtual screening and inhibitor design to identify more potent inhibitors targeting the allosteric pocket of PDE5 (hydrogen bond analysis was included in Supporting Information).

We summarize here the structure–activity relationship of the hits as follows (see Figure 4 and Table S1): (1) A scaffold with two to four fused rings is essential for inhibitors targeting the allosteric pocket of PDE5. (2) A hydrogen bond acceptor, such as carbonyl or methoxyl, is crucial for interacting with the key residue His617. (3) A single two-fused-ring structure without any chain or ring substituents is almost inactive. (4) The presence of two or more rotatable single bonds linking the two-fused-ring scaffold to an aromatic ring typically reduces PDE5 affinity. (5) A bulky nonplanar substituent group, such as 1,3-dioxolane is tolerated only when attached to a four-fused-ring scaffold.

## 4. Materials and Methods

### 4.1. Molecular Modelling

In the first step, a pharmacophore model was developed to efficiently screen the small-molecule database SPECS, significantly reducing its size. Next, molecular docking was conducted to provide an initial prediction of binding modes and energies between the protein and the molecules, further narrowing down the dataset. Finally, molecular dynamics (MD) simulations combined with the molecular mechanics/Generalized Born (MM-GBSA) method were utilized to achieve a more accurate prediction of binding modes and their corresponding binding energies. The final dataset consisted of 33 molecules with favorable binding patterns and the highest binding energies, which were subsequently evaluated through enzymatic bioassays.

#### 4.1.1. Database Preparation

The database SPECS (http://www.specs.net, accessed on 1 December 2023), which contains a great number of small molecules (197,788), was applied in our virtual screening and filtered by “Rule of Five” [29] using Molecular Operating Environment (MOE 2010) when generating multiple conformations, constituting the initial dataset as **Dataset 01**. A maximum of 255 conformations for each compound were generated using the “Conformation Import” method in MOE.

#### 4.1.2. Pharmacophore Modelling and Screening

Crystal structures of PDE5 bound to the inhibitor **(*S*)-7e** [25] were used to construct a robust 3D pharmacophore model for screening **Dataset 01**. The co-crystal structure (PDB ID: 6VBI) was downloaded from the Protein Data Bank and utilized to develop the pharmacophore model. The spatial positions and interactions between the ligand and protein were thoroughly analyzed, identifying common ligand features such as aromatic rings, hydrophobic centers, and hydrogen bond donor and acceptor groups as pharmacophore elements. For one model, the excluded volume of amino acid residues was not applied, while in the other model, the excluded volume was included as a filtering feature. These pharmacophore models were then used to filter compounds in **Dataset 01**, resulting in the creation of **Dataset 02** and **Dataset 03** for each pharmacophore model, respectively.

#### 4.1.3. Molecular Docking Screening

False positive compounds (PAINS) in virtual screening can interfere with other detection methods, resulting in false hits or misleading lead compounds. To address this, PAINS screening was conducted to eliminate potential false positives, leading to the creation of **Dataset 04**.

Molecular docking using Surflex-dock embedded in Sybyl-X1.2 [33] was conducted on the compounds in **Dataset 04**, as previously reported (see Supporting Information) [26], and those with higher docking scores and favorable binding patterns were selected to form **Dataset 05**. Notably, it was essential for the critical residue His617 in the allosteric pocket to form at least one hydrogen bond with the target compound. For the PDE5 protein, hydrogen atoms were added using the H++ web server (http://newbiophysics.cs.vt.edu/H++/, accessed on 10 January 2024), and the protonation states were appropriately adjusted.

#### 4.1.4. Molecular Dynamics-Assisted Screening

MD simulations were subsequently performed to more accurately predict the binding patterns of the molecules. The software AMBER 16.0 [31] was used to simulate the interactions between the molecules in **Dataset 05** and PDE5. Partial atomic charges for the docking poses of the molecules were calculated using the Hartree−Fock method at the 6-31G* level with Gaussian 03 [34]. The Antechamber program was then employed to fit the restricted electrostatic potential (RESP) and assign parameters from the general AMBER force field (GAFF). The amber03 force field was used for the protein, and His617 in the allosteric pocket was modeled as HID (protonated at the δ-N position). An 8 Å TIP3P water box in the shape of a truncated octahedron, along with Na^+^ ions, was added to neutralize the system.

The well-prepared ligands and proteins were subjected to MD simulations. A 20 ns MD simulation was performed in the *NPT* ensemble, maintaining a constant pressure of 1 atm and a temperature of 300 K. Periodic boundary conditions were applied, with an 8 Å cutoff for long-range electrostatic interactions using the particle mesh Ewald (PME) method [35]. The SHAKE algorithm [36] was used to constrain all bonds involving hydrogen atoms, allowing a time step of 2 fs. To accelerate the simulations, an Intel(R) Xeon(R) Gold 6150 CPU and an NVIDIA Tesla V100 GPU were utilized, leveraging GPU capabilities for efficient floating-point calculations. From the initial 4 ns of the trajectory, 100 snapshots were extracted for MM-PBSA and MM-GBSA binding free energy calculations [32], which were conducted with default parameter settings. A similar calculation was performed on the 100 snapshots extracted from the final 4 ns of the trajectory (see Supporting Information). The entropy contribution of the PDE5–ligand complexes was omitted to expedite the calculations.

Following MD simulations, the compounds in **Dataset 05** with top-ranked negative binding energies and visually inspected optimal binding patterns were selected to form **Dataset 06**. Finally, all compounds in **Dataset 06** were purchased from SPECS and evaluated in an enzymatic bioassay to obtain their inhibitory activity against PDE5.

### 4.2. Bioactivity Tests

#### 4.2.1. Protein Expression and Purification

The cDNA encoding the catalytic domain of human PDE5A1 was generated through site-directed mutagenesis of the bovine PDE5A gene, as previously described [37]. The coding region for amino acids 535–860 of PDE5A1 was amplified by PCR and subcloned into the pET15b expression vector. The resulting plasmid, pET-PDE5A1, was then introduced into E. coli strain BL21 (CodonPlus) for overexpression. The *E. coli* cells carrying pET-PDE5A1 were grown in lysogeny broth (LB) medium at 37 °C to an optical density (A600) of 0.7, followed by induction with 0.1 mM isopropyl β-D-thiogalactopyranoside and further growth at 15 °C overnight. Recombinant PDE5A1 was purified using a Ni-NTA affinity column (Qiagen), followed by thrombin cleavage to remove the His-tag. Further purification was performed using Q-Sepharose and Sephacryl S300 columns (Amersham Biosciences). A typical purification batch yielded more than 10 mg of PDE5A1 with >95% purity from a 2 L cell culture. Similar protocols were adopted for the protein expression and purification of PDE10A [28]. PDE6C and PDE11A were purchased from BPS Bioscience.

#### 4.2.2. In Vitro Assay for Potential PDE5 Inhibitors

The enzymatic activities were measured using ^3^H-cAMP or ^3^H-cGMP as substrates, following previously reported methods [28,37]. Briefly, the wild-type proteins were incubated with a reaction mixture containing 20 mM Tris–HCl (pH 7.5), 10 mM MgCl_2_, 0.5 mM dithiothreitol (DTT), and ^3^H-cAMP or ^3^H-cGMP (20–40k cpm/assay) at room temperature for 15 min. The reaction was stopped by adding 0.2 M ZnSO_4_. The reaction product, ^3^H-AMP or ^3^H-GMP, was precipitated by adding 0.2 N Ba(OH)_2_, while unreacted ^3^H-cAMP or ^3^H-cGMP remained in the supernatant. Radioactivity in the supernatant was measured using a liquid scintillation counter. Inhibition was assessed at two concentrations of inhibitors, with appropriate substrate and enzyme concentrations. Sildenafil, a PDE5 inhibitor, was used as a positive control to validate the assay conditions.

## 5. Conclusions

This study presents a molecular dynamics-assisted approach for discovering novel PDE5 inhibitors targeting a unique allosteric pocket, combining pharmacophore model screening, molecular docking, MD simulations, and bioassay. Thirty-three compounds from the commercial SPECS database were selected through screening, and seven of them demonstrated moderate inhibition against PDE5, with inhibition ratios exceeding 50% at a concentration of 10 μM. Additionally, seven distinct scaffold types were identified. Among these, **AI-898/12177002** showed a promising IC_50_ value of 1.6 μM and more than 10-fold selectivity towards PDE6. Binding analysis revealed two crucial hydrogen bonds with His617 and Ser766. Unfortunately, due to its low solubility in water, we failed to cocrystallize **AI-898/12177002** with PDE5 to validate its binding mode. However, based on the binding mode predicted by MD simulations, various hydrophilic substituents can be attached to the scaffold to further enhance solubility and increase selectivity over PDE6 and PDE11. It should also be noted that **AI-898/12177002** effectively inhibits PDE10 at a micromolar level, which needs to be addressed in the upcoming structural optimization. Nonetheless, the strategy employed in this study strikes a balance between computational efficiency and enhancing the hit ratio, highlighting the rational discovery and design of allosteric PDE5 inhibitors with fewer side effects.

## Data Availability

The data presented in this study are available within the article and Supplementary Materials.

## Supplementary Materials

The following supporting information can be downloaded at https://www.mdpi.com/article/10.3390/molecules30030588/s1, Figure S1: The established pharmacophore models; Figure S2: The binding modes of representative molecules with PDE5 based on molecular docking; Table S1: The predicted binding free energies of the 33 purchased compounds and their inhibition ratios against PDE5 and PDE10; Table S2: Tanimoto coefficients of the hits compared to reported PDE5 inhibitors.
