# Supplementary material for: Molecular Dynamics-Assisted Discovery of Novel Phosphodiesterase-5 Inhibitors Targeting a Unique Allosteric Pocket
Source: Molecules. 2025 Jan 27;30(3):588. doi: 10.3390/molecules30030588 (PMC11820102; doi:10.3390/molecules30030588)
Supplement: Supplementary file 1 [file molecules-30-00588-s001.zip › molecules-3399227-supplementary.pdf]

Supporting Information

Table of Contents:

1. Details on pharmacophore modelling and screening…………………………………………S2

2. Figure S1. The established pharmacophore models……………………………………….S3

3. Details on molecular docking……………………………………………………………...S4

4. Figure S2. The binding modes of representative molecules with PDE5 based on molecular docking……..…………………………………………………………………….………..S5

5. Table S1. The predicted binding free energies of the 33 purchased compounds and their inhibition ratios against PDE5 and PDE10………………………………………………………………..S7

6. Details on binding free energy calculations…………………………………………….....S11

7. Hydrogen bond analysis during MD simulations………………………………………..S12

8. Table S2. Tanimoto coefficients of the hits compared to reported PDE5 inhibitors……………..S14

1. Details on pharmacophore modelling and screening

For each pharmacophore model, features were defined as spheres with varying radii, centered on the rings or atoms of the inner ligand **(*S*)-7e** located in the allosteric pocket of PDE5. For the initial model (Model 1), four conjugated hydrophobic ring features, namely Hyd1, Hyd2, Hyd3, and Hyd4, were defined in the A, B, C, and D rings, respectively, with radii of 1 Å. Two hydrogen bond acceptor/donor features, namely Don1/Acc1 and Don2/Acc2, were defined in the oxygen atoms of methoxy and carbonyl groups, respectively, with radii of 1 Å (Figure S1A and S1B). During pharmacophore screening, three out of four features representing hydrophobic centers were selected, and a binary choice was made between the hydrogen bond donor/acceptor features for partial matching. A total of 26,310 compounds in Dataset 01 passed the pharmacophore screening, which is an excessive number. We adjusted the radii of Hyd4, Don1/Acc1, and Don2/Acc2 to 0.7 Å in the initial pharmacophore

model, resulting in Model 2 (Figure S1C). Using Model 2 for pharmacophore screening, 13,620 compounds in **Dataset 01** successfully passed the screening. To further refine the dataset, the conformations in **Dataset 02** were filtered using Model 3, a pharmacophore model based on Model 2 with excluded volume constraints for neighboring residues to prevent steric hindrance with the allosteric pocket (Figure S1D). This step significantly reduced the dataset size from 13,620 compounds in **Dataset 02** to 1,862 compounds in **Dataset 03**.

2. Figure S1. The established pharmacophore models

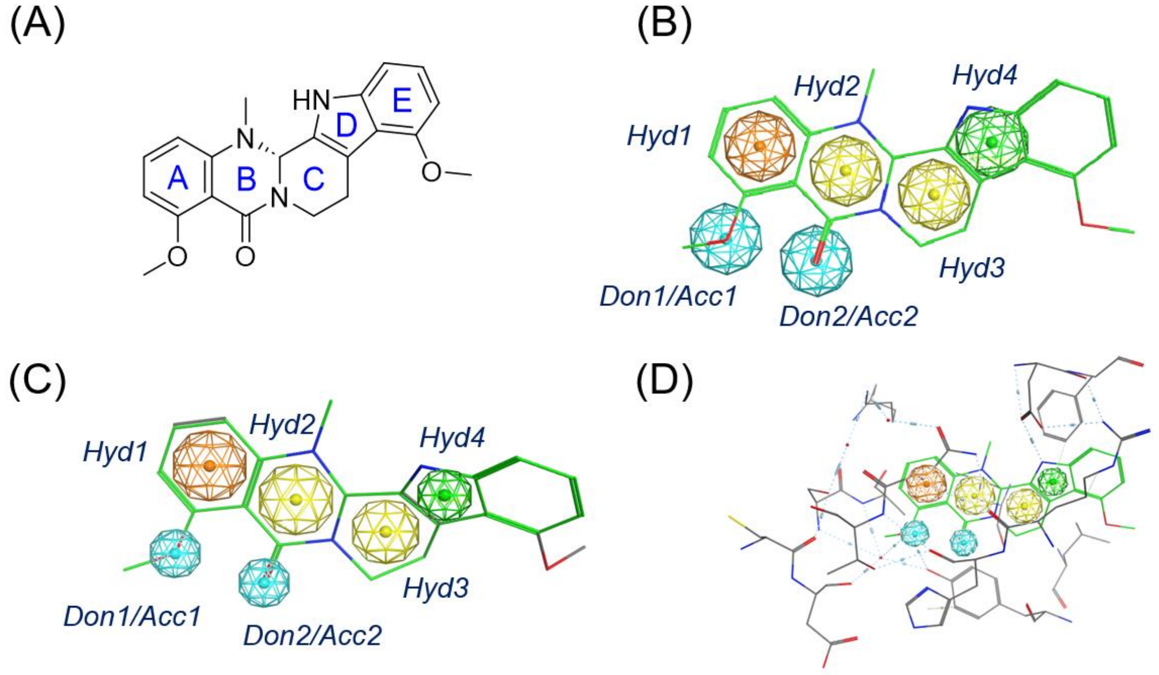

Figure S1. The established pharmacophore models.

3. Details on Molecular Docking

The crystal structure of PDE5 complexed with a highly selective inhibitor **(*S*)-7e** (PDB ID: 6VBI) was used in this study. Molecular docking was conducted using Surflex-dock, a component of Tripos Sybyl (version X2.0, Tripos Software, Inc., El Cerrito, CA). Hydrogen atoms were added, and the ionizable residues were protonated to reflect neutral pH conditions. The protomol represents a group of molecular fragments selected from CH_4, C=O, and N−H, and was placed in PDE5's allosteric pocket by identifying empty 1 Å voxels between marked residues using the bound ligand as the reference. It was then automatically optimized locally by 3D Gaussian smoothing for placement, with high-scoring fragments retained, constituting the docking site. The parameters proto_thresh and proto_bloat indicate the extent to which the protomol can be buried within the protein and the permissible outward extension beyond the cavity, respectively. The proto_thresh was set to 0.5, while the proto_bloat was designated as 0. After preparing the protomol, molecular docking was conducted. The parameter of "Maximum Number of Poses per Ligand" was set to 10, resulting in at most 10 top-ranked docked conformations retained. CScore calculations were performed to obtain docking scores between each conformation and PDE5. All designed molecules were docked to the prepared PDE5 protein, and the top 97 molecules possessing both higher docking scores and suitable binding patterns were selected for subsequent MD simulations, followed by binding free energy calculations.

4. Figure S2. The binding modes of representative molecules with PDE5 based on molecular docking

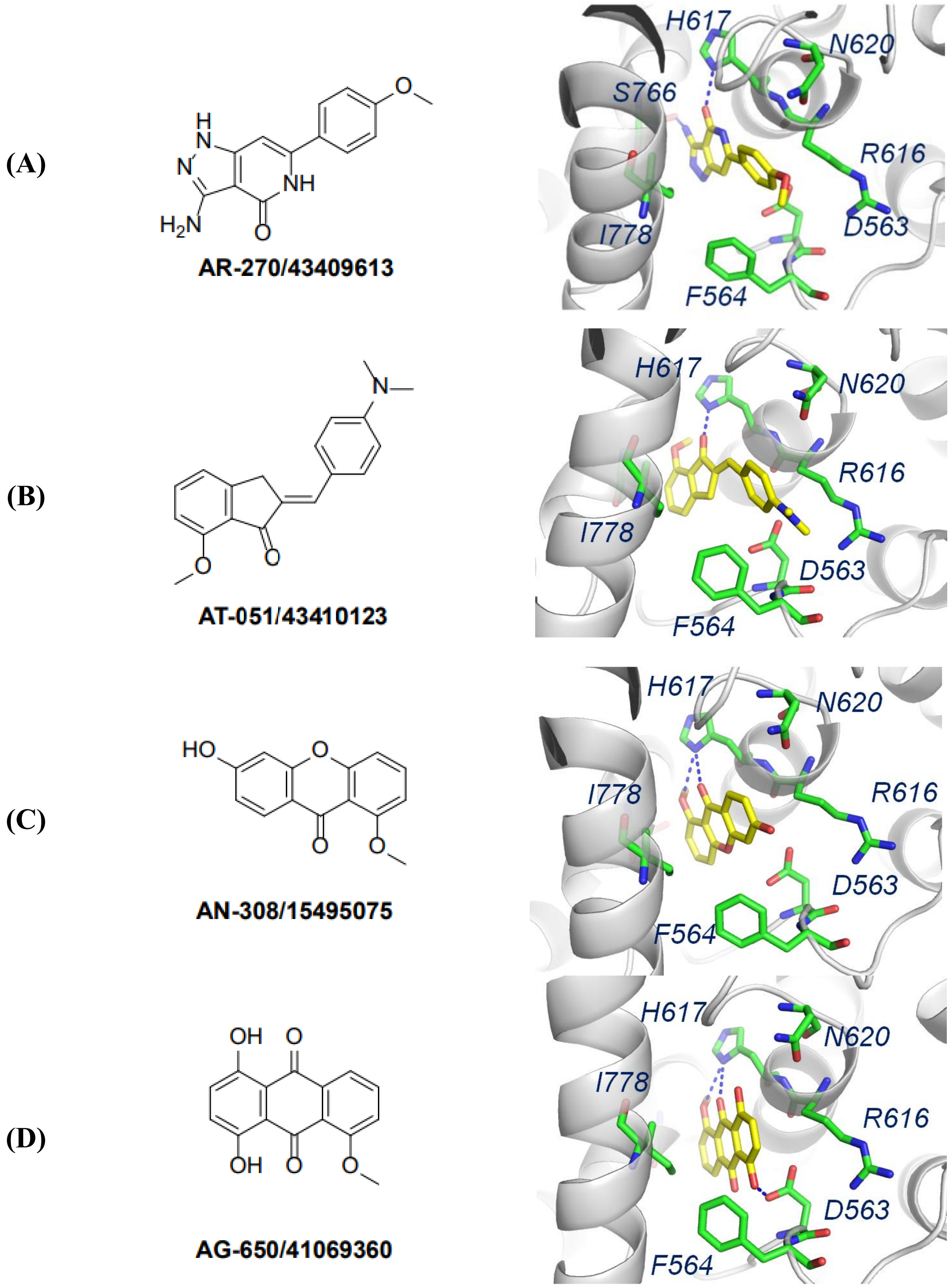

(E)

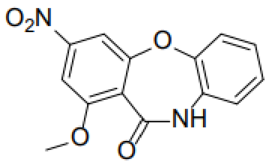

AG-205/40649212

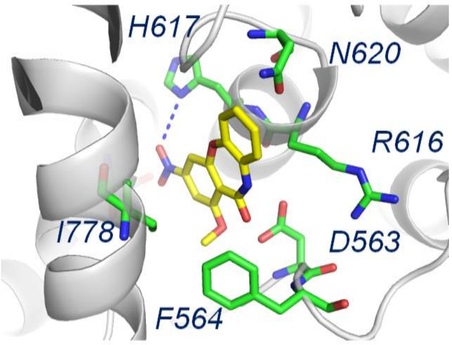

(F)

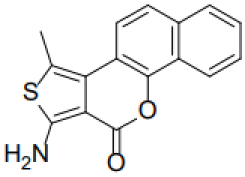

AI-898/12177002

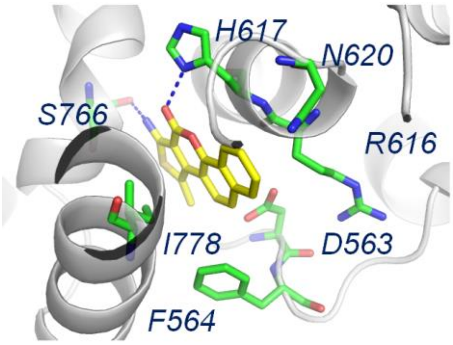

(G)

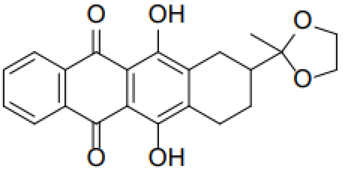

AE-508/36401018

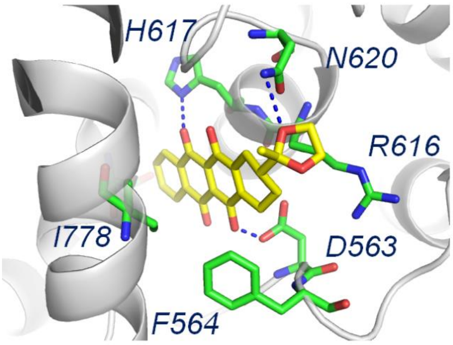

Figure S2. The binding modes of representative molecules with PDE5 based on molecular docking.

5. Table S1. The predicted binding free energies of the 33 purchased compounds and their inhibition ratios against PDE5 and PDE10.

SPECS No.	Structure	Inhib. Ratio [a]	Inhib. Ratio [b]	ΔG_{pred} [c] (kcal/mol)	ΔG_{pred} [d] (kcal/mol)
AB-321/ 43115524	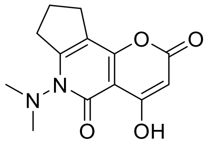	0 [e]<br>0 [f]	n.d. [g]	-28.39 ± 3.46 [j]<br>-27.97 ± 5.40 [k]	-35.17 ± 2.65 [l]<br>-34.67 ± 3.34 [m]
AB-323/ 13887209	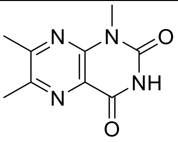	8%<br>3%	n.d.	-14.22 ± 4.24<br>-7.24 ± 5.14	-18.57 ± 2.46<br>-14.02 ± 2.86
AB-323/ 13887271	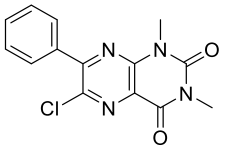	28%<br>6%	n.d.	-17.83 ± 4.06<br>-19.23 ± 4.17	-27.07 ± 2.87<br>-25.01 ± 2.59
AE-406/ 41056104	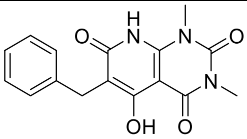	17%<br>0	n.d.	-26.48 ± 3.10<br>-20.91 ± 3.67	-32.98 ± 2.54<br>-27.39 ± 2.77
AE-406/ 41056516	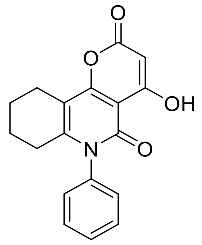	3%<br>15%	n.d.	-19.57 ± 4.29<br>-23.86 ± 3.28	-28.37 ± 2.53<br>-26.76 ± 2.29
AE-406/ 41056559	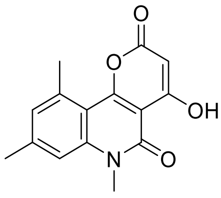	6%<br>0	n.d.	-28.18 ± 3.18<br>-25.66 ± 2.90	-31.68 ± 2.33<br>-30.60 ± 2.16
AE-406/ 41056975	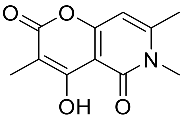	10%<br>0	n.d.	-19.99 ± 4.61<br>-15.65 ± 4.49	-28.72 ± 3.01<br>-23.84 ± 2.83
AE-473/ 12326003	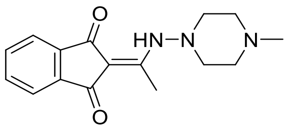	6%<br>9%	n.d.	-18.91 ± 3.37<br>-27.81 ± 4.41	-29.16 ± 2.31<br>-33.84 ± 2.89
AE-508/ 36401018	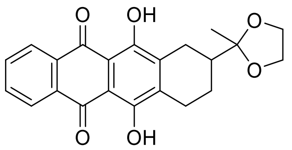	62%<br>14%	72% [h]<br>9% [i]	-27.99 ± 3.78<br>-23.45 ± 2.65	-35.52 ± 2.86<br>-26.45 ± 2.25

AE-641/<br>06374028	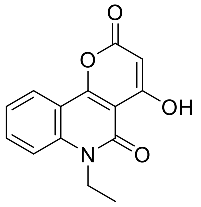	41%<br>13%	n.d.	-15.77 ± 3.67<br>-19.29 ± 4.35	-19.00 ± 2.69<br>-20.75 ± 2.84
AE-641/<br>11289091	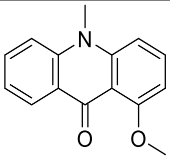	11%<br>0	n.d.	-27.46 ± 3.45<br>-23.36 ± 4.17	-36.97 ± 2.40<br>-35.65 ± 2.66
AE-646/<br>37121059	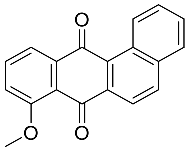	5%<br>7%	n.d.	-25.86 ± 3.35<br>-23.51 ± 3.17	-31.95 ± 2.23<br>-30.53 ± 2.41
AE-848/<br>32306056	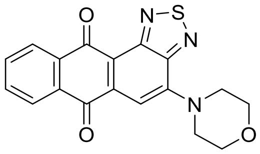	4%<br>0	n.d.	-23.12 ± 3.93<br>-28.46 ± 4.35	-36.92 ± 2.77<br>-38.76 ± 2.55
AF-399/<br>42447040	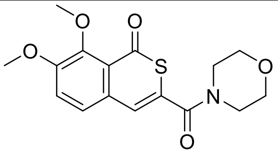	0<br>0	n.d.	-19.48 ± 2.58<br>-18.33 ± 3.60	-23.71 ± 2.04<br>-23.84 ± 3.28
AF-886/<br>31107054	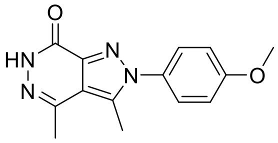	11%<br>0	n.d.	-28.05 ± 3.07<br>-27.78 ± 3.15	-33.59 ± 2.59<br>-32.47 ± 2.68
AG-205/<br>40649212	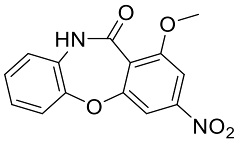	50%<br>0	86%<br>1%	-15.47 ± 5.15<br>-17.82 ± 3.48	-23.54 ± 2.63<br>-23.37 ± 2.74
AG-650/<br>41069241	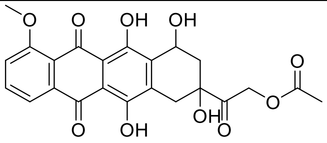	29%<br>8%	n.d.	-25.00 ± 4.37<br>-28.56 ± 3.87	-29.07 ± 2.97<br>-34.88 ± 3.73
AG-650/<br>41069360	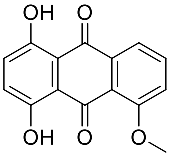	52%<br>25%	81%<br>43%	-19.80 ± 4.30<br>-13.50 ± 4.39	-29.19 ± 2.82<br>-28.71 ± 2.46
AH-262/<br>34335035	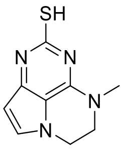	21%<br>10%	n.d.	-16.76 ± 3.30<br>-16.85 ± 4.13	-30.06 ± 2.72<br>-24.38 ± 2.01

AI-621/<br>37187006	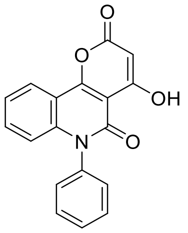	37%<br>19%	n.d.	-18.90 ± 4.19<br>-16.22 ± 3.02	-32.76 ± 3.46<br>-21.66 ± 2.53
AI-898/<br>12177002	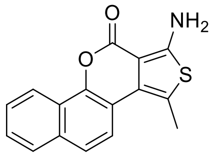	95%<br>12%	79%<br>24%	-26.06 ± 3.68<br>-19.94 ± 3.34	-30.42 ± 3.45<br>-31.78 ± 2.49
AJ-292/<br>40884632	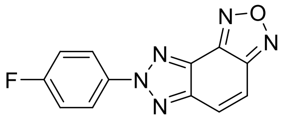	20%<br>0	n.d.	-21.72 ± 3.95<br>-15.92 ± 3.91	-26.72 ± 1.91<br>-23.60 ± 1.90
AN-308/<br>15495075	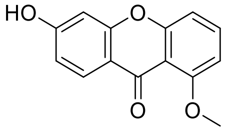	62%<br>4%	93%<br>50%	-26.01 ± 3.20<br>-26.13 ± 3.33	-34.84 ± 2.01<br>-34.91 ± 2.07
AO-289/<br>42804228	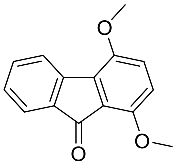	38%<br>0	n.d.	-23.25 ± 3.14<br>-23.59 ± 2.91	-33.05 ± 2.18<br>-32.48 ± 2.00
AO-476/<br>41055966	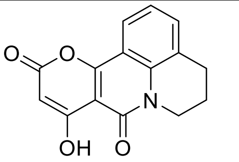	0<br>0	n.d.	-15.24 ± 5.38<br>-22.41 ± 4.62	-25.89 ± 2.23<br>-29.65 ± 2.62
AO-476/<br>43250037	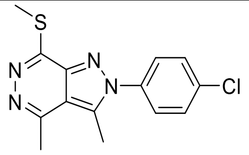	17%<br>0	n.d.	-27.83 ± 4.20<br>-25.67 ± 3.46	-36.54 ± 3.24<br>-34.12 ± 2.87
AO-476/<br>43417581	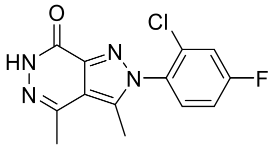	5%<br>0	n.d.	-18.49 ± 3.75<br>-23.52 ± 3.41	-26.10 ± 2.87<br>-29.80 ± 2.46
AP-501/<br>41557792	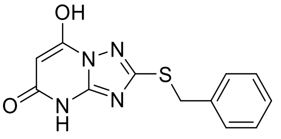	5%<br>0	n.d.	-17.40 ± 3.56<br>-17.14 ± 3.47	-30.33 ± 2.46<br>-31.47 ± 2.27
AP-782/<br>41885349	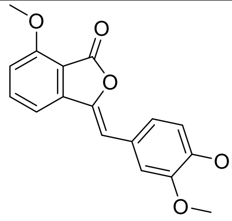	37%<br>0	n.d.	-24.28 ± 3.02<br>-19.76 ± 3.94	-33.79 ± 2.61<br>-35.01 ± 2.20

AP-906/<br>42288215	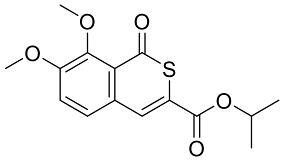	25%<br>0	n.d.	-19.12 ± 4.16<br>-17.21 ± 4.31	-29.31 ± 2.82<br>-25.59 ± 3.12
AQ-390/<br>41344097	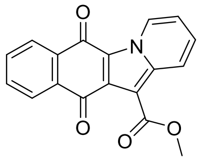	20%<br>0	n.d.	-23.21 ± 4.49<br>-23.54 ± 3.43	-30.52 ± 2.43<br>-32.59 ± 2.26
AR-270/<br>43409613	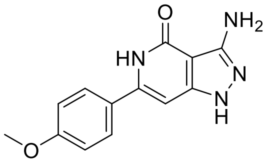	67%<br>11%	23%<br>6%	-20.08 ± 2.93<br>-14.98 ± 3.91	-31.61 ± 2.68<br>-30.31 ± 2.80
AT-051/<br>43410123	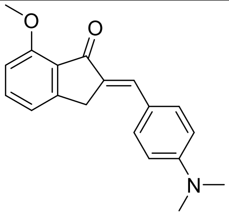	59%<br>7%	42%<br>2%	-25.20 ± 4.01<br>-24.70 ± 3.98	-36.99 ± 2.27<br>-35.77 ± 2.52

[a] Inhibition ratio against PDE5A (535–860) at 10 μM and 1 μM concentrations. Sildenafil was used as a positive control with over 50% inhibition ratio at 10 nM concentrations.

[b] Inhibition ratio against PDE10A (449-770) at 10 μM and 1 μM concentrations. Papaverine was used as a positive control with around 50% inhibition ratio at 50 nM concentrations.

[c] Binding free energies predicted by MM-PBSA and MM-GBSA methods, based on the first 4 ns of the 20 ns molecular dynamics simulation results.

[d] Binding free energies predicted by MM-PBSA and MM-GBSA methods, based on the last 4 ns of the 20 ns molecular dynamics simulation results.

[e] Inhibition ratio at a 10 μM concentration.

[f] Inhibition ratio at a 1 μM concentration.

[g] n.d.: not determined.

[h] Inhibition ratio at a 10 μM concentration.

[i] Inhibition ratio at a 1 μM concentration.

[j] Binding free energies predicted by MM-PBSA methods.

[k] Binding free energies predicted by MM-GBSA methods.

[l] Binding free energies predicted by MM-PBSA methods.

[m] Binding free energies predicted by MM-GBSA methods.

6. Details on binding free energy calculations

In light of the MM-PBSA and MM-GBSA method, the binding free energy (ΔG_{bind}) can be calculated by the following Eq. 1, where the free energies of complex, receptor and ligand are represented by G_{comp}, G_{rec} and G_{lig}, respectively.

$$\Delta G_{bind} = G_{comp} - G_{rec} - G_{lig} \qquad (1)$$

The binding free energy of each system was evaluated as the sum of the MM energy (E_{MM}), the solvation free energy (G_{solv}), and the entropy contribution (S), respectively, as described in Eq. 2.

$$\Delta G_{bind} = \Delta E_{MM} + \Delta G_{solv} - T\Delta S \qquad (2)$$

ΔE_{MM} is the gas phase interaction energy, which can be decomposed into $E_{MM,comp}$, $E_{MM,rec}$ and $E_{MM,lig}$. Solvation free energy is evaluated by the sum of the electrostatic solvation free energy (ΔG_{PB}) and nonpolar solvation free energy ($\Delta \mathrm{G}_{np}$), resulting in Eq. 3.

$$\Delta G_{solv} = \Delta G_{PB} + \Delta \mathrm{G}_{np} \qquad (3)$$

ΔG_{PB} was calculated by the Poisson Boltzmann (PB) Eq, whereas ΔG_{np} was calculated according to Eq. 4. The default parameters were adopted, with $\gamma = 0.0072$ kcal/(Å^2) and $b = 0$ kcal/mol.

$$\Delta G_{np} = \gamma \text{ SASA} + b \qquad (4)$$

For a compromise between efficiency and accuracy, the entropy contribution term ($-T\Delta S$) was omitted for ΔG_{bind} in Eq. 2, since the calculations of the entropy contribution are extremely time-consuming for large protein-ligand systems.

7. Hydrogen bond analysis during MD simulations

Three representative hits were selected for hydrogen bond analysis based on the 20 ns MD trajectories using the "cpptraj" program in Amber16. For **AE-508/36401018**, two hydrogen bonds are commonly observed with Asp563, which appears in 76% of the snapshots from the entire trajectory, and Asn620, which appears in 40% of them (Figure S3A). For **AI-898/12177002**, two hydrogen bonds are commonly observed with His617, which appears in 77% of the snapshots from the entire trajectory, and Ser766, which appears in 34% of them (Figure S3B). For **AT-051/43410123**, only one hydrogen bond is commonly observed with His617, which appears in 86% of the snapshots from the entire trajectory (Figure S3C). In most other hits, a hydrogen bond with His617 is also frequently observed throughout the MD trajectories (data not shown). Thus, a hydrogen bond with His617 is essential for PDE5 affinity for most hits.

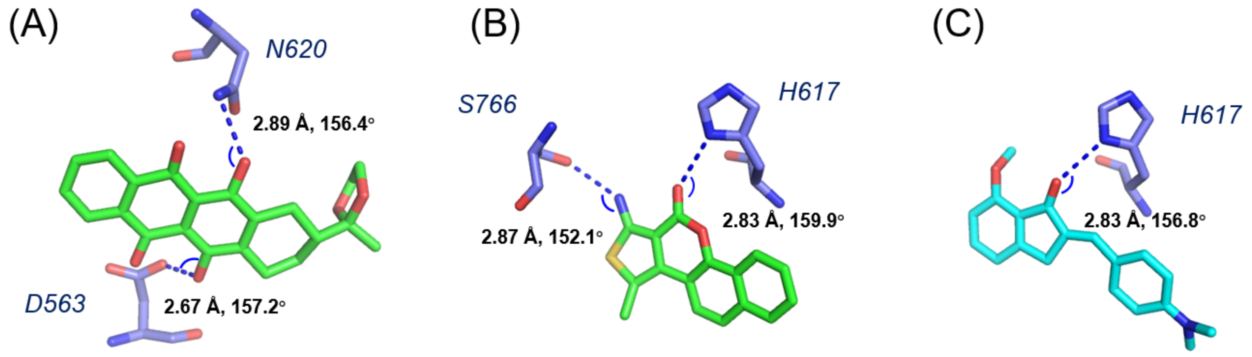

Figure S3. The average distances and angles of hydrogen bonds between the hits and residues in the PDE5 allosteric pocket during the 20 ns MD simulations. (A) **AE-508/36401018**; (B) **AI-898/12177002;** (C) **AT-051/43410123**.

To ensure a robust evaluation of the interactions, we extracted the snapshot after 20 ns MD simulations retaining the hit and concerned residues, and performed structural optimization using the B3LYP/6-31G(d) method in Gaussian 03. Then, interaction (hydrogen bond) energy calculations for **AE-508/36401018**-PDE5 and **AI-898/12177002**-PDE5 using the counterpoise correction method to eliminate basis set superposition error (BSSE) (E(interaction) = E(complex) + E(BSSE) – E(protein) – E(ligand)). These calculations focused on key residues (His617, Ser766, Asp536, and Asn620) and the hits using the MP2/6-31G(d) method implemented in Gaussian 03. Data indicates that **AI-898/12177002** can form strong hydrogen bonds with His617, exhibiting an interaction energy of -10.65

kcal/mol. In contrast, the hydrogen bonding with Ser766 is relatively weaker, with an interaction energy of -1.21 kcal/mol. Similar data can be seen with the **AE-508/36401018**-PDE5 complex, which shows a weaker hydrogen bond energy of -2.93 kcal/mol with Asn620 and a stronger energy of -14.56 kcal/mol with Asp563.

8. Table S2. Tanimoto coefficients of the hits compared to reported PDE5 inhibitors [a]

SPECS No.	Structure	Maximum Tanimoto coefficient
AE-508/36401018	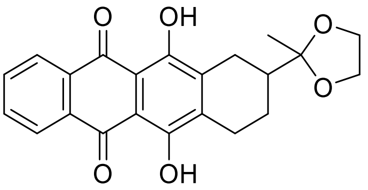	0.30
AG-205/40649212	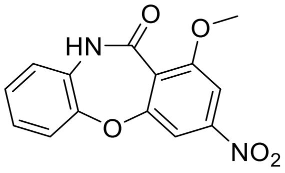	0.31
AG-650/41069360	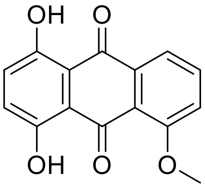	0.28
AI-898/12177002	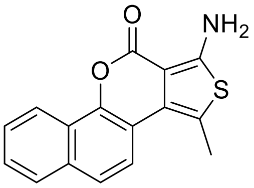	0.27
AN-308/15495075	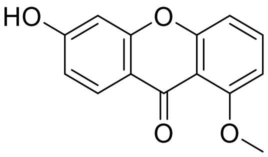	0.21
AR-270/43409613	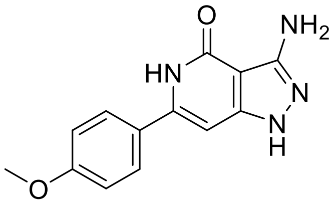	0.33
AT-051/43410123	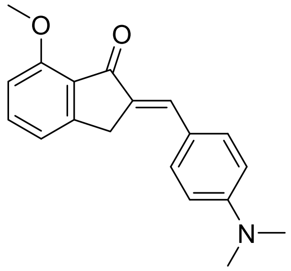	0.32

[a] A total number of 2,179, collected from databases of ChEMBL and BindingDB, with affinities no more than 10 μM.
